# Endoscopic treatment for a hematoma-mediated colon obstruction caused by acupuncture: A rare case report

**DOI:** 10.1055/a-2078-0392

**Published:** 2023-05-10

**Authors:** Shiwei Li, Siyu Sun, Gouxin Wang

**Affiliations:** Department of Gastroenterology, Shengjing Hospital of China Medical University, Shenyang, China

A 54-year-old man experienced abdominal pain, nausea, and vomiting for 3 days after abdominal acupuncture 15 days before. The lab results were unremarkable. Upon computed tomography (CT), a high-density intraluminal mass consistent with hemorrhage was revealed to completely obstruct the colon ( Fig. 1 ). Colonoscopy demonstrated a complete lumen obstruction caused by an extensive purple hematoma in the transverse colon.

**Fig. 1:**
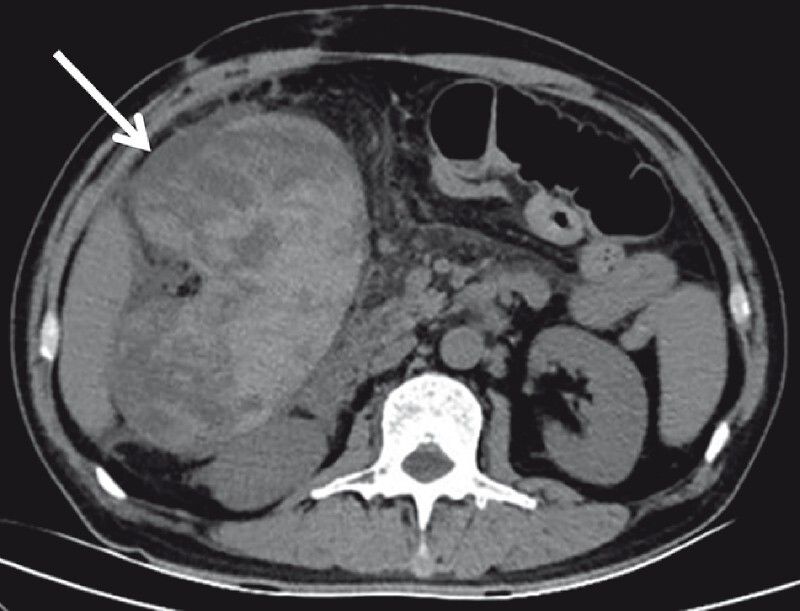
A preoperative abdominal computed tomography (CT) indicating complete colonic obstruction with high-density contents consistent with bleeding.

The treatment plan prioritized endoscopic treatment of an intramural hematoma over surgical intervention. The top of the hematoma was carefully punctured with an electroacupuncture knife, followed by an incision. A large amount of liquefied, pale-yellow substance was observed emanating from the hematoma upon puncture. When the endoscope was inserted into the incision, the intestinal tissue was found to have a honeycomb-like adhesion. This procedure was repeated at multiple points on the hematoma. The hematoma was then accessed through a larger incision; an attempt was made to release the adhering tissue. A portion of the tissue was removed using electrocautery, and the hematoma’s interior was flushed to ensure that there were no bleeding perforations in the bowel wall. The hematoma was successfully decompressed using this procedure ( Video 1 ).

**Video 1** Endoscopic incision and drainage for the treatment of obstruction caused by colonic hematomas.


The patientʼs symptoms improved following conservative treatment. A CT taken 7 days later revealed partial improvement in the colonic obstruction and decreased density in the colon proper ( Fig. 2 ). A CT scan 53 days later indicated that the colonic stricture and entire hematoma had completely resolved ( Fig. 3 ).

**Fig. 2:**
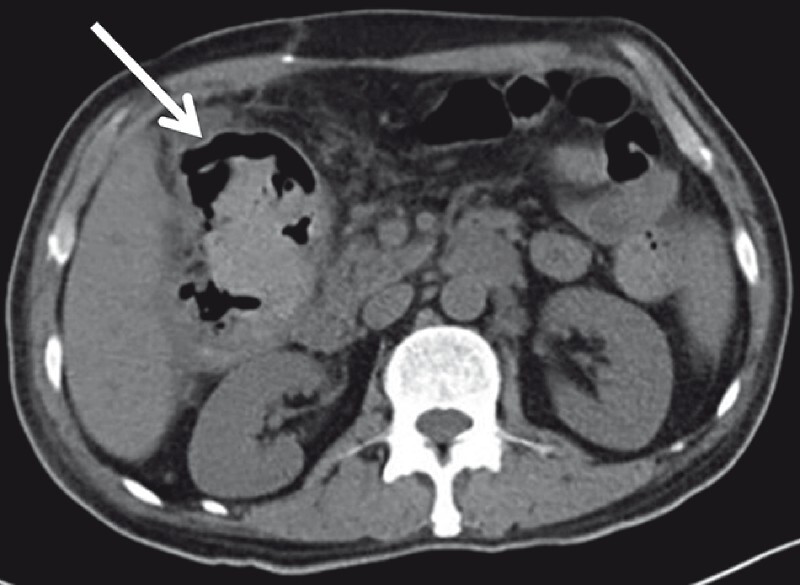
A CT taken 7 days post-operation, revealing partial improvement in the colonic obstruction and decreased density in the colon proper.

**Fig. 3:**
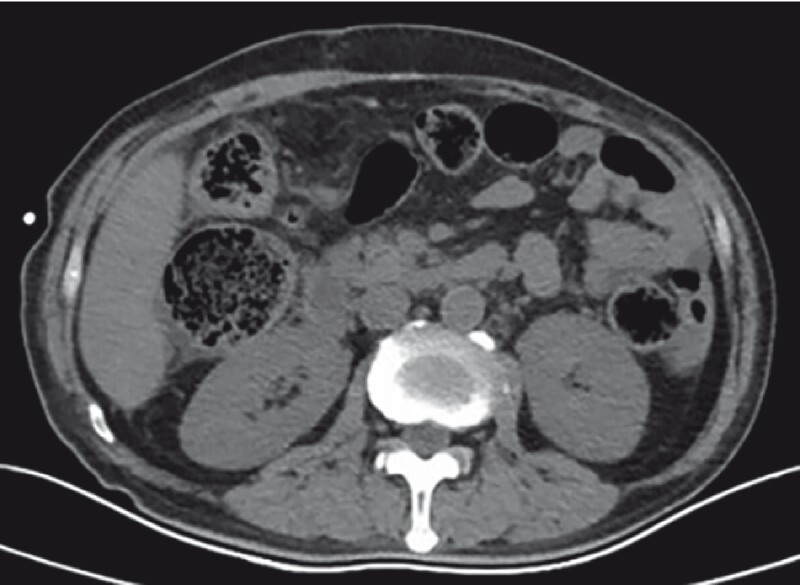
A CT scan taken 53 days after endoscopic treatment, showing the colonic stricture and entire hematoma almost entirely resolved.

Intramural hematomas are a rare condition caused by various etiologies 1 . We demonstrated the efficacy of endoscopic incision and drainage for treating an obstruction caused by colonic hematomas. There have been previous reports of this method being used to treat esophageal or duodenal hematomas 2 3 4 . However, the procedure’s complications need to be further assessed in prospective controlled trials before it can be widely recommended.

Endoscopy_UCTN_Code_TTT_1AQ_2AJ

## Notes

Endoscopy E-Videos

https://eref.thieme.de/e-videos
*E-Videos* is an open access online section of the journal *Endoscopy* , reporting on interesting cases and new techniques in gastroenterological endoscopy. All papers include a high-quality video and are published with a Creative Commons CC-BY license. Endoscopy E-Videos qualify for HINARI discounts and waivers and eligibility is automatically checked during the submission process. We grant 100% waivers to articles whose corresponding authors are based in Group A countries and 50% waivers to those who are based in Group B countries as classified by Research4Life (see: https://www.research4life.org/access/eligibility/ ). This section has its own submission website at https://mc.manuscriptcentral.com/e-videos
