# Correction: Endoscopic treatment for a hematoma-mediated colon obstruction caused by acupuncture: A rare case report

**DOI:** 10.1055/a-2092-1486

**Published:** 2023-05-17

**Authors:** Shiwei Li, Siyu Sun, Guoxin Wang

**Affiliations:** Department of Gastroenterology, Shengjing Hospital of China Medical University, Shenyang, China

**Correction**

**Correction: Endoscopic treatment for a hematoma-mediated colon obstruction caused by acupuncture: A rare case report** Li S, Sun S, Wang G et al. Endoscopic treatment for a hematoma-mediated colon obstruction caused by acupuncture: A rare case report. Endoscopy 2023 (S1), 55: E713–E714, doi:10.1055/a-2078-0392 In the above-mentioned article, the name of Guoxin Wang has been corrected. This was corrected in the online version on May 15, 2023.

